# Advanced diagnostic endoscopy in the upper gastrointestinal tract: Review of the Japan Gastroenterological Endoscopic Society core sessions

**DOI:** 10.1002/deo2.359

**Published:** 2024-04-10

**Authors:** Hiroya Ueyama, Toshiaki Hirasawa, Tomonori Yano, Hisashi Doyama, Hajime Isomoto, Kazuyoshi Yagi, Takashi7 Kawai, Kenshi Yao

**Affiliations:** ^1^ Department of Gastroenterology Juntendo University School of Medicine Tokyo Japan; ^2^ Department of Gastroenterology Cancer Institute Hospital Japanese Foundation for Cancer Research Tokyo Japan; ^3^ Department of Gastroenterology, Endoscopy Division National Cancer Center Hospital East Chiba Japan; ^4^ Department of Gastroenterology Ishikawa Prefectural Central Hospital Ishikawa Japan; ^5^ Division of Gastroenterology and Nephrology Tottori University Faculty of Medicine Tottori Japan; ^6^ Department of Gastroenterology Niigata University Local Medical Care Education Center Uonuma Kikan Hospital Niigata Japan; ^7^ Department of Gastroenterological Endoscopy Tokyo Medical University Hospital Tokyo Japan; ^8^ Department of Endoscopy Fukuoka University Chikushi Hospital Fukuoka Japan

**Keywords:** advanced diagnostic endoscopy, image‐enhanced endoscopy, artificial intelligence, endocytoscopy, endoscopic ultrasound

## Abstract

The Japan Gastroenterological Endoscopy Society (JGES) held four serial symposia between 2021 and 2022 on state‐of‐the‐art issues related to advanced diagnostic endoscopy of the upper gastrointestinal tract. This review summarizes the four core sessions and presents them as a conference report. Eleven studies were discussed in the 101st JGES Core Session, which addressed the challenges and prospects of upper gastroenterological endoscopy. Ten studies were also explored in the 102nd JGES Core Session on advanced upper gastrointestinal endoscopic diagnosis for decision‐making regarding therapeutic strategies. Moreover, eight studies were presented during the 103rd JGES Core Session on the development and evaluation of endoscopic artificial intelligence in the field of upper gastrointestinal endoscopy. Twelve studies were also discussed in the 104th JGES Core Session, which focused on the evidence and new developments related to the upper gastrointestinal tract. The endoscopic diagnosis of upper gastrointestinal diseases using image‐enhanced endoscopy and AI is one of the most recent topics and has received considerable attention. These four core sessions enabled us to grasp the current state‐of‐the‐art in upper gastrointestinal endoscopic diagnostics and identify future challenges. Based on these studies, we hope that an endoscopic diagnostic system useful in clinical practice is established for each field of upper gastrointestinal endoscopy.

## INTRODUCTION

The Japan Gastroenterological Endoscopy Society (JGES) held four serial symposia on state‐of‐the‐art issues related to advanced diagnostic endoscopy of the upper gastrointestinal tract. These were as follows (Table 1):

- 101st core session: Advanced diagnostic endoscopy: Challenges and prospects of upper gastroenterological endoscopy; moderators, Kazuyoshi Yagi and Hajime Isomoto.
- 102nd core session: Advanced upper gastrointestinal endoscopic diagnosis for decision‐making on therapeutic strategy; moderators, Kenshi Yao and Takashi Kawai.
- 103rd core session: Advanced diagnostic endoscopy: AI·CAD; moderators, Tomonori Yano and Toshiaki Hirasawa.
- 104th core session: Advanced diagnostic endoscopy: Evidence and new developments related to the upper gastrointestinal tract; moderators, Hisashi Doyama and Hiroya Ueyama.

**TABLE 1:** The chairpersons and topics of each Japan Gastroenterological Endoscopy Society (JGES) Congress.

Congress	Chairpersons	Topics	Number of research presentations
101st	Hajime Isomoto (Division of Gastroenterology and Nephrology, Tottori University Faculty of Medicine), Kazuyoshi Yagi (Department of Gastroenterology, Niigata University Local Medical Care Education Center, Uonuma Kikan Hospital)	Advanced diagnostic endoscopy: Challenges and future prospects of upper gastroenterological endoscopy	11
102nd	Takashi Kawai (Department of Gastroenterological Endoscopy, Tokyo Medical University Hospital, Tokyo, Japan), Kenshi Yao (Department of Endoscopy, FukuokaUniversity Chikushi Hospital, Fukuoka, Japan)	Advanced upper GI endoscopic diagnosis for decision‐making on therapeutic strategy	10
103rd	Tomonori Yano (Endoscopy division, Department of Gastroenterology National Cancer Center Hospital East, Chiba, Japan), Toshiaki Hirasawa (Departments of Gastroenterology Cancer Institute Hospital of the Japanese Foundation for Cancer Research, Tokyo, Japan)	Advanced diagnostic endoscopy: AI·CAD	8
104th	Hiroya Ueyama (Department of Gastroenterology, Juntendo University School of Medicine, Tokyo, Japan), Hisashi Doyama (Department of Gastroenterology, Ishikawa Prefectural Central Hospital, Kanazawa, Japan)	Advanced diagnostic endoscopy: Evidence and new developments in the upper gastrointestinal tract	12

### Topics of discussion in the JGES core sessions

A total of 41 research presentations were conducted and various discussions were held on these topics. The research presentations in the four core sessions are listed in Table 2. Discussions in the four core sessions are summarized in the Supporting Information (Text S1). When the contents of the research presentations were categorized according to the organ and target disease, endoscopic diagnosis of gastric cancer was the most frequently reported procedure with 18 presentations (43.9%), followed by endoscopic diagnosis of duodenal neoplasia with 10 presentations (24.4%), endoscopic diagnosis of esophageal cancer with six presentations (14.6%), endoscopic diagnosis of pharyngeal cancer with 1 presentation, and other diagnostic analysis in six presentations (14.6%). Among the 18 research presentations on gastric cancer, 11 were on image‐enhanced endoscopy (IEE; narrow band imaging [NBI], blue light imaging [BLI], texture and color enhancement imaging [TXI], and linked color imaging [LCI]) and five were on artificial intelligence (AI). Among the 10 research presentations on superficial non‐ampullary duodenal epithelial tumors (SNADETs), eight were on diagnostic strategies and two were on AI. Additionally, among the five research presentations on esophageal cancer, three were on IEE and two were on AI. Other than the above, the presentations were unique and novel for various diseases. Owing to space limitations, we will only summarize selected topics from these sessions.

**TABLE 2:** The research presentations reported at each Japan Gastroenterological Endoscopy Society (JGES) Congress.

Congress	Stomach	Duodenum	Esophagus	Others
101st	Gastric cancer: 1. NBI versus WLI 2. ECS versus M‐NBI 3. ECS + AI 4. TXI versus WLI Gastric neoplasia: PDD + 5‐ALA AIG: cast‐off skin appearance	Duodenal epithelial neoplasia: 1. Simple scoring system 2. M‐NBI with acetic acid	Esophageal cancer: type B2 Eosinophilic esophagitis: beige mucosa Esophageal varices: RDI	none
102nd	Gastric cancer: 1. M‐NBI on H.pylori status 2. RSGL in H.pylori‐uninfected patients 3. Histological type 4. 1200N (OLYMPUS) 5. TXI in ECS + AI Intragastric pressure	Duodenal epithelial neoplasia: 1. Diagnostic algorithm 2. ECS 3. non‐biopsy versus biopsy, M‐NBI	Esophageal cancer: ECS	none
103rd	Gastric cancer: 1. AI (depth of invasion) 2. AI (depth of invasion) Anatomical location: AI (diagnosis) Group 2 lesion: AI + molecular maker	Duodenal epithelial neoplasia: AI (detection)	Esophageal cancer 1. AI (detection) 2. AI (detection)	SEL in Upper GI: AI (diagnosis)
104th	Gastric cancer: 1. Surveillance endoscopy 2. TXI versus WLI versus NBI 3. TXI versus WLI 4. Blood flow rate analysis system 5. M‐BLI/NBI versus biopsy	Duodenal epithelial neoplasia: 1. Simple scoring system 2. M‐AANBI 3. Diagnostic algorithm WLI + M‐NBI 4. ECS + AI	Esophageal cancer: LCI versus BLI BEA: M‐AANBI ve M‐NBI	Diagnostic algorism of superficial pharyngeal cancer
Total	Twenty‐one research presentations	Ten research presentations	Eight research presentations	Two research presentations

Abbreviations: 5‐ALA, 5‐aminolevulinic acid; BEA, Barrett's esophageal adenocarcinoma; BLI, Blue laser imaging; ECS, endocytoscopy; LCI, Linked Color Imaging; M‐AANBI, acetic acid with M‐NBI; M‐NBI, magnifying endoscopy with NBI; NBI, narrow band imaging; PDD, photodynamic diagnosis; RDI, red dichromatic imaging; RSGL, raspberry‐like, reddish‐elevated lesion; SEL, subepithelial lesions; TXI, texture and color enhancement imaging; WLI, white light imaging.

### Current status of advanced diagnostic endoscopy for gastric lesions

During the core sessions, various topics related to the detection and diagnosis of early gastric cancer were discussed. Recently, difficulties in the endoscopic detection and diagnosis of *Helicobacter pylori*‐negative early gastric cancer, which is currently showing an increased incidence, have occasionally been reported.^1^, ^2^, ^3^, ^4^ There have been many reports on the usefulness of IEE, including the LCI and BLI technologies of FUJIFILM and the NBI and TXI technologies of OLYMPUS, and AI systems in overcoming this problem.

Ono et al.^5^ reported that LCI was more effective than white light imaging (WLI) in detecting neoplastic lesions in the pharynx, esophagus, and stomach when studied in a controlled, multicenter, randomized trial.^5^ The detection rates of LCI and WLI were 5.5% and 3.3%, respectively. Dohi et al.^6^ reported that the real‐time detection rate of early gastric cancer with primary BLI‐bright was significantly higher than that with primary WLI (93.1% vs. 50.0%; *p* < 0.05). Therefore, LCI and BLI‐bright improve the real‐time early gastric cancer detection rate compared to WLI. Yoshida et al.^7^ detected early gastric cancer in 44 (1.9%) and 53 (2.3%; *p* = 0.412) patients in the WLI and second‐generation NBI (2G‐NBI) groups, respectively, during primary esophagogastroduodenoscopy (EGD). 2G‐NBI did not improve real‐time early gastric cancer detection rate compared with WLI. However, the positive predictive value of 2G‐NBI was significantly higher than that of WLI. Regarding the endoscopic diagnosis of early gastric cancer, Ezoe et al.^8^ reported that the diagnostic ability of magnifying endoscopy with NBI (M‐NBI, 90.4 %) and WLI + M‐NBI (96.6 %) was significantly better than that of WLI (64.8). Hence, M‐NBI and WLI + M‐NBI improve diagnostic performance compared with WLI in early gastric cancer. Dohi et al.^9^ also reported that the sensitivity of magnifying endoscopy with BLI (M‐BLI) for diagnosis was significantly higher than that of WLI (93.8 vs. 46.9%), as was its specificity (91.6 vs. 80.0%), positive predictive value (78.9 vs. 44.1%), negative predictive value (97.7 vs. 81.7%), and accuracy (92.1 vs. 71.2%). Therefore, M‐BLI enhances diagnostic performance compared with WLI in early gastric cancer. Some reports have described the usefulness of TXI in detecting gastric neoplasms and improving the visibility of early gastric cancer.^10^, ^11^, ^12^, ^13^, ^14^, ^15^, ^16^ However, randomized controlled trials showing the usefulness of TXI in detecting early gastric cancer during screening endoscopy have not been conducted.

Recently, several AI systems for gastric cancer exhibiting robust performance have been reported.^17^, ^18^, ^19^, ^20^, ^21^, ^22^, ^23^, ^24^ The first AI‐based endoscopic computer‐aided detection (CADe) system for gastric cancer was reported by Hirasawa et al.^17^ in 2018. The sensitivity for detecting gastric cancer per image analysis was 92.2 %; however, the positive predictive value was low (30.6%). Ikenoyama et al.^18^ reported that the AI showed a higher sensitivity than endoscopists (58.4% vs. 31.9%), whereas it showed lower specificity (87.3% vs. 97.2%) and positive predictive value (26.0% vs. 46.2%). A few prospective studies comparing the performance of CADe in gastric cancer between endoscopists and CADe systems have also been performed. Luo et al.^19^ first conducted a multicenter case‐control study evaluating the performance of a CADe system in upper gastrointestinal tract cancers. They reported an accuracy of 92.7% in detecting both gastric and esophageal cancers. The diagnostic performance of the CADe system was comparable to that of endoscopists with >10 years of experience (94.2% vs. 94.5% sensitivity) and superior to that of competent (85.8%) and trainee (72.2%) endoscopists. Wu et al.^20^ reported on a computer‐assisted detection/diagnosis (CAD) system for gastric neoplasms called ENDOANGEL, which was created based on deep reinforcement learning and convolutional neural network (CNN) and can be used for various purposes including the detection of gastric cancer and precancerous lesions, differentiation of cancerous and noncancerous lesions, prediction of tumor invasion depth, and anatomical detection of the upper gastrointestinal tract. The miss rate in the CAD‐first group (AI‐first, *n* = 907) was significantly lower than that in the routine EGD group (routine‐first, *n* = 905; 6.1% vs. 27.3%, *p* = 0.015). Moreover, Li et al.^21^ and Ueyama et al.^22^ reported a high diagnostic accuracy for their computer‐assisted diagnosis (CADx) systems for gastric cancer based on M‐NBI images, with sensitivities of 95.4% and 98%, and specificities of 71.0% and 100%, respectively. Wu et al.^23^ prospectively evaluated the performance of ENDOANGEL, which was developed using M‐NBI images and reported a sensitivity of 100% and a specificity of 82.54%, which were equivalent to those of endoscopists. Most reports on AI systems for gastric cancer have shown that AI has a better diagnostic performance than non‐expert endoscopists and that their performance is equivalent to that of experts.^25^ However, further studies are required to evaluate the usefulness of endoscopic AI systems in clinical settings. Currently, in clinical practice, AI systems are expected to have high sensitivity but low false‐positive rates. New AI systems and further analyses are needed to reduce the false‐positive rates while maintaining sensitivity.

Although some IEEs, such as LCI and M‐NBI, have been clinically introduced for the diagnosis of gastric cancer, there are still many limitations to AI diagnosis. Although the prevalence of gastric cancer is low in Europe and the United States in particular, the detection rate of early gastric cancer is also expected to be low. Further research on the detection of early gastric cancer, including the development of new modalities such as AI, in foreign countries is needed.

### Current status of advanced diagnostic endoscopy for duodenal lesions

With the recent increase in the rate of SNADET detection, significant progress has been made in endoscopic diagnosis and treatment. However, there remains a need to improve the quality of endoscopic diagnosis and provide more curative and safer treatments. Therefore, the differentiation of SNADETs from non‐neoplastic lesions is being developed.^26^, ^27^, ^28^, ^29^ According to Nakayama et al.,^26^ when considering each superficial structure, it was best to evaluate the combination of white opaque substance and light blue crest as a superficial duodenal epithelial tumor (SDET) with an open‐loop structure, and the combination of demarcation line and enlarged marginal epithelium as an SDET with a closed‐loop structure. For SDET diagnosis, the sensitivity, specificity, and accuracy were at 88.4%, 98.3%, and 92.2%, respectively. Yamasaki et al.^27^ reported that 88% (62/70) of SNADET surface patterns were pit‐type, whereas 79% (35/44) of non‐neoplasm surface patterns were groove‐type. Their diagnostic algorithm for differentiating SNADETs from non‐neoplasms showed high sensitivity (96%) and specificity (95%) in the descending and horizontal duodenum. Ishii et al.^29^ developed a scoring system to identify Vienna Classification C4/5 lesions. In their analysis, a tumor diameter of 10–19 mm (odds ratio [OR], 3.81; 95% confidence interval [CI], 1.02–14.2; *p* = 0.04) or ≥20 mm (OR, 95.2; 95% CI, 10.4–871.0; *p* < 0.001), red color (OR, 14.5; 95% CI, 3.55–59.6; *p* < 0.001), and the presence of an irregular surface pattern (OR, 12.4; 95% CI, 3.00–51.4; *p* < 0.001) or an irregular vessel pattern (OR, 13.7; 95% CI, 4.03–46.6; *p* < 0.001) were significant independent predictors of Vienna Classification C4/5. The diagnostic accuracy, sensitivity, and specificity were at 92%, 95%, and 93%, respectively. In addition, in the studies by Akazawa et al.^30^ and Toya et al.,^31^ SNADETs showed distinct endoscopic and clinicopathological features when considering the mucin phenotype. Therefore, tumor location, color, macroscopic type, and endoscopic findings, including those observed by M‐NBI, are useful for distinguishing the mucin phenotypes of SNADETs.^30^ Additionally, the pinecone pattern observed under magnifying endoscopy with crystal violet staining may be a characteristic feature of gastric SNADETs, especially pyloric gland adenoma.^31^

With regard to AI systems, Inoue et al.^32^ reported that a trained CNN detected 94.7% (378 of 399) of SNADETs on an image basis (94% [280 of 298] of adenomas, 100% [101 of 101] of high‐grade dysplasia, and 100% of SNADETs on a tumor basis). However, there are relatively few reports on AI systems related to SNADETs, which could be considered a challenge for the future.

Various diagnostic criteria and strategies for SNADETs have been reported, but the terminology has not been uniform, and an integrated classification has not been established. To solve these problems, the unification of terminology and the establishment of a diagnostic system through multicenter studies are required.

### Current status of advanced diagnostic endoscopy for esophageal lesions

The endoscopic detection and diagnosis of esophageal squamous cell carcinoma has been widely studied, and the usefulness of WLI, NBI, BLI, and Lugol's, among others, in detection and diagnosis is well established, including in daily clinical practice.^33^, ^34^, ^35^, ^36^, ^37^, ^38^, ^39^ However, compared with the accuracy of type B1 and type B3 vessels for T1a‐EP (epithelial)/LPM (lamina propria mucosae) and T1b‐SM (submucosa) 2, the accuracy of type B2 vessels for T1a‐MM (muscularis mucosae) /T1b‐SM1 is relatively low.^39^ Tanaka et al.^40^ reported that the invasion depth (EP/LPM: MM/SM1: SM2) of B2 in an area with a diameter <4 mm (B2‐Narrow) or ≥4 mm (B2‐Broad) was 46:11:1 and 1:15:4, respectively. B2‐Broad had a sensitivity, specificity, positive predictive value, and negative predictive value of 61%, 98%, 95%, and 79%, respectively, thereby enabling the prediction of T1a‐MM or deeper invasion. The diagnostic accuracy of type B2 was improved by determining its area. Although many problems related to the detection and diagnosis of esophageal squamous cell carcinoma, including in‐depth diagnosis, have been solved, further analysis using more accurate diagnostic tools is needed.

Barrett's esophagus (BE) is a metaplastic condition secondary to gastroesophageal reflux disease. BE is also a precursor of esophageal adenocarcinoma, which, although rare in Japan, is one of the most common cancers in Western countries. However, the prevalence of gastroesophageal reflux disease has increased significantly over the past few decades in Japan, possibly leading to an incremental increase in BE. Given that the associated inherent risk of adenocarcinoma endoscopic identification of early neoplasms is still not sufficiently reliable or subjective, using conventional endoscopy for targeted biopsy is extremely difficult. Over the last decade, acetic acid enhancement and NBI combined with magnification endoscopy have enabled the identification of early neoplasms.^41^, ^42^ The primary feature detected by conventional WLI is a reddish area or lesion located on the right anterior wall. IEE, including dye‐based (chromoendoscopy with dye solutions such as indigo carmine, methylene blue, crystal violet, etc.) or equipment‐based techniques (NBI, autofluorescence imaging), and the acetic acid‐spraying method have been applied to detect or characterize Barrett's neoplasia.^41^, ^42^, ^43^, ^44^, ^45^, ^46^, ^47^, ^48^ IEE may be useful in characterizing tumors and diagnosing lateral tumor extensions.

A systematic review and meta‐analysis reported that in 14 studies (1590 patients) that assessed the use of AI in the endoscopic diagnosis of esophageal squamous cell carcinoma, the pooled sensitivity and specificity were 91.2% (84.3–95.2) and 80% (64.3–89.9), respectively.^49^ In addition, nine studies (478 patients) that evaluated AI capabilities in diagnosing esophageal adenocarcinoma showed a pooled sensitivity and specificity of 93.1% (86.8–96.4) and 86.9% (81.7–90.7), respectively. Despite promising results, the application of AI in real‐time endoscopy is limited, and further multicenter trials are required to accurately assess its use in routine practice.^50^, ^51^, ^52^

As mentioned above, accurate endoscopic diagnosis of squamous cell carcinoma can be achieved in clinical practice using IEE. However, the endoscopic detection and diagnosis of Barrett's adenocarcinoma may be difficult, and new equipment‐based techniques and further research are required.

## CONCLUSIONS

During these core sessions, various studies evaluating endoscopic diagnosis using IEE and AI in the field of upper gastrointestinal endoscopy were discussed. Based on these studies and recently published research articles, we hope that an endoscopic diagnostic system that is useful in clinical practice is established for each field of upper gastrointestinal endoscopy.

## CONFLICT OF INTEREST STATEMENT

Hajime Isomoto has previously received a research grant out of this work from FUJIFILM.

Tomonori Yano received honoraria for a research grant out of this work from FUJIFILM, OLYMPUS, HOYA PENTAX, and Ambu.

Hisashi Doyama received consulting fees from Otsuka Pharmaceutical, Otsuka Pharmaceutical Factory, Eisai, EA Pharma, AstraZeneca, and Olympus.

Toshiaki Hirasawa received honoraria for lectures from AI medical service.

Tomonori Yano received honoraria for lectures from FUJIFILM and OLYMPUS.

Hajime Isomoto is a DEIC of Digestive Endoscopy.

The other authors declare no conflict of interest.

## Supporting information



Text S1 101st–104th Congress of the Japan Gastroenterological Endoscopy Society Core Session.
