# Supplementary material for: Advanced diagnostic endoscopy in the upper gastrointestinal tract: Review of the Japan Gastroenterological Endoscopic Society core sessions
Source: DEN Open. 2024 Apr 10;4(1):e359. doi: 10.1002/deo2.359 (PMC11004903; doi:10.1002/deo2.359)
Supplement: Supplementary file 1 — Text S1 101st–104th Congress of the Japan Gastroenterological Endoscopy Society Core Session. [file DEO2-4-e359-s001.docx]

[101st Congress of the Japan Gastroenterological Endoscopy Society Core Session]

Chairpersons:

Hajime Isomoto (Division of Gastroenterology and Nephrology, Tottori University Faculty of Medicine)

Kazuyoshi Yagi (Department of Gastroenterology, Niigata University Local Medical Care Education Center, Uonuma Kikan Hospital)

The 101st Congress of the Japan Gastroenterological Endoscopy Society upper section core session (symposium) addressed the topic of “Advanced Diagnostic Endoscopy: Challenges and Prospects of Upper Gastroenterological Endoscopy.” A summary of the event is presented here. The presenters have been omitted from the abstract. (1) In a comparative study of white light for early gastric cancer histological diagnosis and narrowband imaging (NBI) magnified observation, no significant difference was found in the correct diagnosis rate. However, the difference in specificity was significant for NBI-magnified observations. (2) Endoscopists were significantly more capable of diagnosing gastric cancer based on endocytoscopy using narrow-band imaging (EC-NBI) rather than the magnified observations obtained by NBI. (3) This result may contribute to the improvement of diagnostic performance in gastric cancer and is beneficial in clinical practice. Upon endocytoscopy, the combined use of methylene blue and crystal violet double staining and texture and color enhancement imaging (TXI) mode improved visibility, enabling the definite diagnosis of post-eradication gastric cancer covered with low atypical epithelium. The combination of artificial intelligence-assisted diagnosis and such novel imaging techniques has improved optical biopsy with endocytoscopy system (ECS) for early gastric cancer. The diagnosis of undifferentiated adenocarcinoma using ECS has been difficult and remains a challenge. (4) TXI mode 1, which emphasizes color tone, unevenness, and brightness, provided greater visibility and showed a large color difference between the inner and outer parts of the lesion compared to white light imaging (WLI). The improved visibility of early gastric cancer with TXI mode 1 compared to WLI is believed to be due to the ability of TXI mode 1 to show a larger color difference between the interior and exterior of the lesions. (5) Photodynamic diagnosis (PDD) using 5-aminolevulinic acid (5-ALA) may help in treating gastric tumors, although its use remains challenging in signet ring cell carcinoma. Currently, the effectiveness of 5-ALA-PDD using LED light is also being verified, and the results will be reported in the future. (6) Autoimmune gastritis (AIG) is characterized by a cast-off skin appearance similar to a magnified image of the normal fundic gland site on the highly atrophic mucosa. The cast-off skin appearance (CSA) finding, as well as the presence of adherent mucus, has been useful in the diagnosis of AIG, and the extent to which this finding adds to WLI-based diagnosis should be investigated in the future. (7) The usefulness of image-enhanced endoscopy (IEE) with narrowband light, such as NBI and blue light imaging (BLI), in screening for superficial esophageal cancer has been well established. In the magnified endoscopy classification of the Japan Esophageal Society, type B2 is defined as an abnormal blood vessel with poor loop formation. However, non-loop blood vessels classified as type B2 include baran blood vessels that do not show submucosal infiltration (hereafter referred to as baran) and B2 blood vessels that can be found in erosions and inflamed areas (hereafter referred to as B2-s). Hence, it is difficult to distinguish true type B2 blood vessels (hereafter referred to as pure B2) that indicate submucosal infiltration. According to the Arima classification, irregularly branched (IB) vessels are known to indicate invasion, which is a typical feature of pure B2 vessels. However, the diagnostic criteria vary among examiners. It would be useful to focus on the following three factors to distinguish IB vessels from non-loop vessels with bifurcation: irregularity of the vessel bifurcation interval/multiple bifurcations of vessels, sharp angular bending of post-bifurcation vessels, and irregularity of the caliber of the post-bifurcation vessels. (8) Beige mucosa is a characteristic feature of eosinophilic esophagitis (EoE), which may be caused by abnormal epithelial differentiation. The beige mucosa is not an appropriate endoscopic indicator of EoE because of its subtle color change but may serve as a marker of histological remission in clinical practice for cases diagnosed with EoE. (9) The new image-enhanced endoscopy technique, red dichromatic imaging (RDI), increased the success rate of intra-injection sclerotherapy for esophageal varices (EV) and decreased the recurrence rate of varicose veins. This functionality enabled the prediction of EV depth and reliable intra-arterial injection, which might have improved the recurrence rate but was not effective in improving the overall survival rate. (10) Treatment guidelines for superficial nonpapillary duodenal epithelial tumors have not yet been established. The need for a preoperative endoscopic diagnostic technique with a high ability to differentiate between low-grade adenoma (LGA) and high-grade adenoma (HGA)/adenocarcinoma (AC) has been increasing given the difference in their biological prognosis. A simple scoring system (SSS) that considers the size, color tone, surface structure as observed by NBI, and abnormal blood vessels may facilitate the endoscopic differentiation between LGA and HGA/AC. (11) Using acetic acid for NBI-magnified observation has helped in distinguishing between adenomas and cancers owing to its ability to detect irregular surface structures in duodenal tumors. As mentioned above, the IEE findings of various diseases of the stomach, esophagus, and duodenum were analyzed, and the development of IEE devices and observation methods was discussed. During the symposium, it became evident that the role of IEE in examinations of the upper gastrointestinal tract will continue to evolve.

[102nd Congress of the Japan Gastroenterological Endoscopy Society Core Session]

Chairpersons:

Takashi　Kawai (Department of Gastroenterological Endoscopy, Tokyo Medical University Hospital, Tokyo, Japan)

Kenshi Yao (Department of Endoscopy, Fukuoka University Chikushi Hospital, Fukuoka, Japan)

Takahasi et al. conducted surveillance using endocytoscopy (EC) for high-risk cases of esophageal squamous cell carcinoma and conducted multicenter prospective studies to evaluate the diagnostic ability of EC for suspected tumor lesions. Screening was conducted using EC, and if suspected lesions of esophageal tumors were observed under white light or during NBI, super-enlarged observation was performed after staining. The EC classification was used for the super-enlarged observation. If a diagnosis of cancer was confirmed, endoscopic submucosal dissection (ESD) was performed. Thirty-seven cases (41 lesions) were diagnosed with cancer and ESD was performed, whereas the tumors in 50 cases that underwent biopsy were found to be non-cancerous. For each lesion, the sensitivity and specificity were 97.6% and 100%, respectively. They concluded that EC is useful in clinical practice for diagnosing suspected esophageal tumors.

Hoteya et al. evaluated the usefulness of magnified endoscopy, based on MESDA-G, for diagnosing early gastric cancer. The diagnosis rates of MESDA-G for uninfected, infected, or post-eradication tumors were 60% (42/70), 96.9% (63/65), and 79.7% (59/74), respectively. In uninfected gastric cancer, the diagnosis rates using MESDA-G for fundic gland-type cancer and signet-ring cell carcinoma were 0% and 44%, respectively. However, these cancers can be diagnosed based on non-magnified endoscopic findings and specific lesion sites. Gastritis-like findings make it difficult to determine the demarcation line (DL) in infected and post-eradication cancers.

Ikeda et al. investigated the endoscopic and clinicopathological features of raspberry-like, reddish, elevated lesions in H. pylori-uninfected stomachs. Raspberry-shaped gastric lesions (RSGLs) were divided into gastric adenocarcinoma of the foveolar type (GAFV), gastric adenocarcinoma of the fundic gland type (GAFG), gastric adenocarcinoma of the fundic gland mucosa type (GAFGM), hyperplastic polyps (HP), and PPI-related polyps with epithelial neoplasia (PPI-L). They were further classified by submucosal tumor shape, redness, and whitish area around the tumor and can also be categorized based on marginal crypt epithelium (MCE) and MESDA-G.

Kanesaka et al. developed an endoscopic diagnostic algorithm for differentiating between the histological types of early gastric cancer. Cases with early gastric cancer (n = 343; cT1, > 10 mm) were enrolled. A diagnosis of differentiated cancer was made if redness was observed under white light and if narrow band imaging (NBI) combined with magnification observation showed a surface microstructure. If the lesion was pale under white light, the patient was diagnosed with undifferentiated cancer. If magnified NBI revealed no microsurface pattern and the microvascular pattern was an open loop the patient was diagnosed with undifferentiated cancer. The use of M-NBI did not improve the accuracy of white-light endoscopy (WLE) in diagnosing depressed/flat undifferentiated-type early gastric cancers (EGCs) but improved its specificity. This may reduce surgical overtreatment by preventing the misdiagnosis of differentiated-type EGC as undifferentiated.

Yanagisawa et al. investigated whether ultrathin transnasal endoscopes can help in diagnosing early gastric cancer. The endoscopic diagnostic sensitivities of 290N and 1200N were 31.8% and 85.7% for white light and 95.5% and 92% for NBI, respectively. At 9%, 1200N has been converted to high definition, and the white light observation ability has greatly improved. After understanding the function of each endoscope, an endoscopic diagnosis can be made even with a nasal endoscope by observing the lesions.

Noda et al. examined the feasibility of improving diagnosis with EC using texture and color enhancement imaging (TXI) by utilizing AI. The area under curve (AUC) for EC gastric cancer detection was 94.1%. With respect to the results of the diagnosis for each image, AI showed excellent specificity compared to endoscopists. The diagnostic match between the two endoscopists had a kappa value of 0.64. Even for each lesion, convolutional neural network (CNN) showed better results than endoscopists in terms of specificity. The numerical rating scale (NRS) in cell atypia was significantly higher with EC examination using TXI (6.9) than with conventional EC examination (6.0).

Imamura et al. aimed to develop an accurate method for measuring intragastric pressure (IP) using endoscopy and determining the pressure required for strong gastric wall extension.

Although it is difficult to accurately determine whether the stomach wall is not extended, the gastric wall may be extended by releasing air into the stomach. For an accurate measurement of gastric pressure, most cases (96%) undergo upper gastrointestinal endoscopy. A strong extension image of the gastric wall was obtained by applying an intragastric pressure of 20 mmHg.

Nakayama et al. developed a diagnostic algorithm for superficial duodenal epithelial tumors (SDETs). Assuming that lesions with surface structures presenting with open-loop structure (OLS) are white opaque substance (WOS)-positive and that lesions with closed-loop structure (CLS) are demarcation line (DL)-positive and enlarged marginal epithelium (EME)-negative, the sensitivity was 88.4%, and tumors and non-tumors could be differentiated with a specificity of 98.3% and a positive diagnosis rate of 92.2%. It was also suggested that OLS may have intestinal traits and CLS may have gastric traits. Based on these results, SDETs diagnostic algorithms were developed, and the diagnosis of SDETs is now possible.

Kubo et al. studied EC diagnosis for superficial non-ampullary duodenal epithelial tumors (SNADETs) to determine treatment indications. By treating non-tumors with 18 or fewer glandular tubes per screen, tumors above 19, and cancerous diseases with a nuclear footprint of 13% or more per screen, lesions of HGA or higher indicated for endoscopic treatment can be identified from EC images. EC for SNADETs can help determine whether endoscopic treatment is indicated to avoid biopsies.

Tsuji et al. investigated whether a previous biopsy leads to overdiagnosis of SNADETs. The results of the non-biopsy and biopsy groups were as follows: sensitivity, 88% (95% Cl: 71.0~96.5) vs. 66% (53.1~76.8), p=0.02; specificity, 95% (77.2~99.9) vs. 89% (76.4~96.4), P = 0.39; positivity rate, 70% (34.8~93.3) vs. 14% (3.0~36.3), p<0.01. For SNADETs in the non-biopsy group, the accuracy of magnified NBI in distinguishing C4/5 lesions from C3 was excellent. The magnified-NBI findings of SNADETs should be evaluated while carefully considering the influence of a previous biopsy.

[103rd Congress of the Japan Gastroenterological Endoscopy Society Core Session]

Chairpersons:

Tomonori Yano (Endoscopy division, Department of Gastroenterology National Cancer Center Hospital East, Chiba, Japan)

Toshiaki Hirasawa (Departments of Gastroenterology Cancer Institute Hospital of the Japanese Foundation for Cancer Research, Tokyo, Japan)

Higashi et al. explored the ability of artificial intelligence (AI) to detect esophageal squamous cell carcinoma (ESCC) in videos of endoscopic examinations. In creating the training video for AI, they used two methods: one annotated with a Bounding Box (AI-B) and the other annotated precisely with semantic segmentation (AI-S). The sensitivity, specificity, and accuracy in detecting ESCC were 100%, 21.9%, and 40.6% for AI-B and 100%, 52.1%, and 63.5% for AI-S, respectively. The Intersection over Union (IoU) was 12.2% for AI-B and 36% for AI-S, indicating that AI-S was significantly more accurate in detecting ESCC. AI, with precise annotation by semantic segmentation to create training images, detected ESCC more accurately and specifically.

Aoyama et al. created an AI model that detects superficial esophageal cancer using endoscopic videos. The patient-based sensitivity, specificity, and accuracy in detecting superficial esophageal cancer were 92.9%, 60.0%, and 71.8%. As for sensitivity by depth, EP (epithelial) was 85.7%, whereas LPM (lamina propria mucosae), MM (muscularis mucosae), and SM (submucosa) 1 were 100%. In conclusion, they constructed an AI model with a high sensitivity for detecting superficial esophageal cancer from videos.

Asanuma et al. developed an AI system that classifies esophagogastroduodenoscopy (EGD) images according to 29 anatomical locations and judges inappropriate endoscopic images based on various aspects such as blurriness, fogginess, lens deposition, halation, remaining fluid or deposits, artifacts, insufficient brightness, and insufficient insufflation. The AI system showed a total accuracy rate of 93.7% and an average sensitivity of 79.2% for anatomical location. However, the AI had a total accuracy rate of 62.8% and an average sensitivity of 53.6% for quality of images. This study demonstrated the potential of AI to contribute to “quality control” in gastric cancer screening.

Watanabe et al. examined the diagnostic ability of a combination of AI-assisted endoscopic diagnosis and molecular markers (p53 mutation, microsatellite markers, and DNA methylation markers) for indefinite dysplastic gastric lesions (Group 2). The optimal area under curve (AUC) was obtained for AI + miR148a (AUC: 0.825). The diagnostic power of the AI + miR148a combination was intermediate between that of board-certified endoscopists (AUC: 0.931) and trainees (AUC: 0.588). This high accuracy may be attributed to the combined benefits of AI in its ability to diagnose the presence, site, and range of the entire lesion and the accuracy of the molecular marker.

Two presentations on the role of AI in the differential diagnosis of the depth of mucosal and submucosal gastric cancers, which remains an important unmet need in clinical practice, were held in succession. Goto et al. established a practical decision rule for the differential diagnosis of the depth of mucosal and submucosal gastric cancer based on cooperation between AI and endoscopists. In this retrospective multicenter study using still images, the authors designed an AI classifier for differentiating between intramucosal and submucosal gastric cancer. They evaluated the ability of AI and endoscopists to differentially diagnose the depth of invasion of gastric cancer. Furthermore, they evaluated the diagnostic ability of several combinations of AI and endoscopists and determined an appropriate combination that provides a better diagnosis than that of endoscopists or AI alone. They concluded that cooperation between AI and endoscopists improved the diagnostic ability for the invasion depth of early gastric cancer.

Yamada et al. developed an AI model for predicting gastric cancer tumor depth and evaluated its diagnostic ability in a retrospective multicenter study. The authors used stored still images with supervised text data of endoscopic and pathological findings as the training dataset. First, they compared five deep learning models to identify the best-performing model in terms of accuracy in detecting deep submucosal invasion of gastric cancer. Moreover, they validated the ability of the AI model through external imaging at another hospital; the AI model showed high accuracy, similar to that of expert endoscopists. They concluded that their AI model demonstrated high accuracy in determining the depth of early gastric cancer and that further evaluation, including a prospective study, is necessary to evaluate the real-life performance of AI.

Niikura et al. developed an AI model for duodenal tumor detection through a retrospective single-center study using still images. Recently, the chances of endoscopic treatment of duodenal tumors have become greater owing to the increasing focus on this issue. The ability to diagnose duodenal tumors is important not only for their detection but also for their differentiation. The authors constructed a prototype AI tool using an object detection model and evaluated the diagnostic ability of the area under the receiver operating characteristic curve (AUC) using a validation set of still images of duodenal tumors and a normal upper GI tract. They achieved better outcomes than those in previous reports. They concluded that they developed a new AI model for duodenal tumor detection and that further validation studies are needed to improve their model.

Hirai et al. investigated the ability of an AI system to classify subepithelial lesions using endoscopic ultrasound (EUS) images. Differential diagnosis of subepithelial tumors is usually difficult, even for experienced endoscopists. They conducted a multi-center retrospective study to develop the AI model using EUS images of subepithelial tumors and used a deep convolutional generative adversarial network (DCGAN) for the training set of uncommon lesions, such as schwannoma, which was the unique aspect of their study. AI performed better than endoscopists in the classification of subepithelial tumors.

In this core session, various studies on the development and evaluation of endoscopic AI in the field of upper gastrointestinal endoscopy were discussed. Most research was conducted in collaboration with engineers in academia or companies. The acceleration of collaborative work with other fields, such as mechanical engineering, should be among the favorable outcomes of the introduction of AI in our field. However, no research has evaluated the ability of AI in real-time because of the absence of a regulatory-approved device for upper gastrointestinal endoscopy. This is a vital limitation of upper endoscopy compared with colonoscopy. Recently, an AI system for helping in the detection and diagnosis of gastric cancer and esophageal squamous cell carcinoma has been approved, and the device was released by FUJIFILM. We hope that AI research expands broadly and that high-quality studies are conducted at the post-marketing stage in the near future.

[104th Congress of the Japan Gastroenterological Endoscopy Society Core Session]

Chairpersons:

Hiroya Ueyama (Department of Gastroenterology, Juntendo University School of Medicine, Tokyo, Japan)

Hisashi Doyama (Department of Gastroenterology, Ishikawa Prefectural Central Hospital, Kanazawa, Japan)

Kikuchi et al. established and evaluated a diagnostic algorithm (0-I, Type B3, 0-IIa+type B2) for the diagnosis of SEP (subepithelial) ≥1000 μm of superficial pharyngeal cancer. In this study, 570 superficial pharyngeal cancer lesions observed using NBI magnification between 2008 and 2021 were analyzed. The accuracy, sensitivity, and specificity of the diagnostic algorithm were 93.7% (531/570), 77.8% (56/72), and 95.4% (475/498), respectively. A diagnostic algorithm established using white light imaging (WLI) and narrow band imaging using magnifying endoscopy (NBI-M) for determining tumor thickness is useful for the risk stratification of lymph node metastasis in pharyngeal cancer.

Ogata et al. investigated the diagnostic performance of blue light imaging (BLI) and linked color imaging (LCI) for esophageal squamous cell carcinoma (ESCC) screening. The detection rates of ESCC in the BLI and LCI groups were 4.0% (14/351) and 4.9% (17/348) (p=0.565), and the miss rates of ESCC were 26.3% (5/19) and 63.3% (19/30) (p = 0.012), respectively. The sensitivity of the diagnostic parameters in the BLI and LCI groups was 75.0% (15/20) and 47.6% (20/42) (p = 0.042), respectively. The diagnostic performance of BLI for ESCC may be superior to that of LCI in patients at a high risk of ESCC; hence, BLI could be considered the standard screening method for ESCC. BLI has an advantage over LCI given the low miss rate and high sensitivity for ESCC. However, BLI was not proven to be superior to LCI in terms of the detection rate of ESCC, which could have been affected by the imbalanced prevalence of ESCC.

Ikenoyama et al. established a classification for Barrett’s esophageal adenocarcinoma (BEA) by combining acetic acid with M-NBI (M-AANBI) and compared the diagnostic ability of M-AANBI for BEA with that of M-NBI. The accuracy, sensitivity, and specificity of BEA diagnosis using M-AANBI and M-NBI were 94.6% vs. 70.4%, 90.8% vs. 64.6%, and 98.5% vs. 76.9%, respectively (p<0.05). The visual analog scale of DL visibility in M-AANBI was significantly higher than that in M-WLI and M-NBI (M-WLI, 3.63; M-NBI, 6.25; M-AANBI, 8.75; P < 0.001). The accuracy for M-NBI alone was significantly improved from 68.5% to 87.3% by combining with acetic acid (M-AANBI) (P<0.05). This study demonstrated that M-AANBI, in addition to M-NBI, may be useful for BEA diagnosis and DL recognition.

Yoshida et al. evaluated newly detected gastric cancer (GC) through surveillance endoscopy after endoscopic observation of the stomach using both 2G-NBI and WLI (intensive endoscopy; I-EGD) as a preplanned secondary analysis of an “early gastric cancer (EGC) detection trial.” The rate of newly detected GC within 15 months of I-EGD was 2.6% (120 lesions). The rate of new GC detected by surveillance endoscopy approximately one year after I-EGD was similar to that of GC detected by I-EGD. The presence of open-type atrophic gastritis (odds ratio 6.00, 95% CI: 2.25-16.01) and EGC detection by I-EGD (odds ratio 4.67, 95% CI: 1.08-20.21) were independent risk factors for new GC detection. Careful endoscopy may be necessary for patients at a high risk of GC, especially those with atrophic gastritis or a history of GC. They concluded that one-year surveillance is warranted for patients at a high risk of GC.

Sakai et al. evaluated the visibility of EGC/adenoma using Texture and Color Enhancement Imaging (TXI), which is an image-enhanced endoscopic technology launched in 2020. The color differences of the gastric tumors were significantly higher with TXI than with WLI (mean ΔE: WLI/NBI/TXI mode 1/mode 2 = 11.5/12.4/18.7/14.7, p<0.001). In addition, the color differences of EGC were significantly greater with TXI than with WLI (mean ΔE: WLI/NBI/TXI mode 1/mode 2 = 11.7/12.3/20.0/15.6, p<0.001). This study demonstrated that TXI may be useful for the detection of EGC, as it could improve the visibility of gastric tumors.

Kemmoto et al. investigated the clinical usefulness of the image-enhanced endoscopy system TXI mode 2 for endoscopic GC screening. The detection rates of GCs, GCs <10 mm, pT1a, and GCs after HP eradication in the TXI mode 2 group were significantly higher than those in the WLI group (0.46% vs. 0.17%, p<0.01; 0.26% vs. 0.07%, p<0.01; 0.39% vs. 0.11%, p<0.01; and 1.4% vs. 0.45%, p<0.01, respectively). They concluded that TXI mode 2 improved the detectability of EGC during endoscopic screening. In particular, TXI mode 2 was useful in the detection of GCs after HP eradication.

Akazawa et al. evaluated the usefulness of an automated blood flow rate analysis system for distinguishing between differentiated-type EGC and patchy redness. They compared the diagnostic ability of the analysis system with that of nine endoscopists. The sensitivity and specificity of EGC diagnosis were 90.3% and 89.7%, respectively, for the automated blood flow rate analysis system and 77.9% and 85.0%, respectively, for the endoscopists (p<0.05). The analysis system showed a better diagnostic performance in terms of differentiating between differentiated-type EGC and patchy redness. Thus, this blood flow rate analysis system may be useful as a new diagnostic modality for EGC.

Yoshida et al. verified that the preoperative histological diagnosis of EGC using ME-BLI/NBI is not inferior to histological diagnosis using biopsy. The positive predictive values of

ME-BLI/NBI and biopsy examination were 96.1% (49/51) and 78.4% (40/51), respectively, and the risk difference was 17.6% [95% CI: 5.2%-30.1%] (p<0.001). The non-inferiority of ME-BLI/NBI was statistically proven in preoperative histological diagnosis compared to biopsy examination. Therefore, for suspected adenocarcinoma lesions, the preoperative histological diagnosis of EGC by ME-BLI/NBI was not inferior to biopsy examinations.

We encountered four presentations on the endoscopic diagnosis of superficial non-ampullary duodenal epithelial tumors (SNADETs), which remains an important issue to be addressed in our practice.

Ishii et al. examined the usefulness of a simple scoring system (SSS) for the differential diagnosis of SNADETs as low-grade adenoma (LGA) or high-grade adenoma (HGA)/adenocarcinoma (AC). They investigated the potential of the SSS by comparing the accuracy rate before and after an educational lecture on SSS. The accuracy, sensitivity, and specificity of the differential diagnosis were 80.8%, 80.7%, and 81.2%, respectively. The accuracy rates before and after the lecture for all lesions, HGA/AC, and LGA were 71%→79%, 70%→81%, and 72％→78%, respectively. This study suggested the SSS as an easy and useful method for diagnosing SNADETs.

Tanaka et al. investigated whether magnifying narrowband imaging with acetic acid (MA-NBI) is useful for differentiating gastric-type (G-type) lesions from intestinal-type (I-type) lesions with regard to SNADETs. The oval surface structure is a specific feature of G-type lesions (tubular/villous/oval structure = G-type: 30/0/70%, I-type: 78/20/2%, p＜0.001); hence, it may be useful for differentiating between the two types of lesions. Regardless of the mucin phenotype, a mixed and irregular surface structure pattern could effectively differentiate category 4/5 from category 3 lesions (mixed = C3: 6%, C4/5: 41%, p=0.003, irregular = C3: 3%, C4/5: 81%, p<0.001). They concluded that MA-NBI may help distinguish the mucin phenotypes of SNADETs and facilitate histological grade diagnosis.

Nakayama et al. explored the endoscopic differences between adenoma and cancer in SNADETs in a prospective study. Cancer lesions were larger than adenoma lesions (32.0 (5-100) mm vs. 16.7 (3-100) mm, p<0.0001), located at the oral side of the duodenum (62.9% vs. 46.5%, p<0.05), and showed elevated morphology compared to adenoma lesions (34.3% vs. 17.5%, p<0.01). When observed by imaged enhanced endoscopy using magnifying endoscopy (IEE-ME), cancer lesions showed a closed-loop structure (58.6% vs. 31.4%, p<0.05), absence of WOS (27.1% vs. 13%, p<0.05), and the presence of irregular microsurface pattern (IMSP) (14.3% vs. 6.7%, p<0.05) and irregular microvascular pattern (IMVP) (25.7% vs. 10.6%, p<0.05) compared to adenoma lesions. The tumor size and absence of WOS were independent predictors of cancer. They concluded that the endoscopic findings of SNADETs differed between cancer and adenoma lesions and that age, tumor size, and presence of WOS were especially important in identifying cancer lesions.

We encountered one presentation on the endoscopic diagnosis of SNADETs using a computer-aided diagnosis (CAD) system.

Kubo et al. created a CAD system for SNADETs using endocytoscopy (EC) and investigated its usefulness for the endoscopic diagnosis of benign lesions, LGA, and HGA/AC. The ratios of methylene blue stained area per screen in benign lesions, LGA, and HGA/AC were 6.75%, 10.1%, and 21.7%, respectively (p<0.001). The median numbers of tubular glands per mm2 in benign lesions, LGA, and HGA/AC were 19, 60, and 125, respectively (p<0.01). Based on these results, they developed a CAD system for differentiating cancer lesions from non-cancer lesions (benign lesions and LGA) based on the percentage of methylene blue-stained areas per screen and the number of tubular glands per mm2 in EC images.

In this core session, various studies evaluating IEE as a means of diagnosis in the field of upper gastrointestinal endoscopy were discussed. Based on these studies, we hope that an endoscopic diagnostic system that is useful in clinical practice is established for each field of upper gastrointestinal endoscopy.
